# Group B *Streptococcus* (GBS) colonization is dynamic over time, whilst GBS capsular polysaccharides-specific antibody remains stable

**DOI:** 10.1093/cei/uxac066

**Published:** 2022-07-08

**Authors:** I L Haeusler, O Daniel, C Isitt, R Watts, L Cantrell, S Feng, M Cochet, M Salloum, S Ikram, E Hayter, S Lim, T Hall, S Athaide, C A Cosgrove, J S Tregoning, K Le Doare

**Affiliations:** St George’s University of London, Paediatric Infectious Diseases Research Group, London, UK; St George’s University of London, Paediatric Infectious Diseases Research Group, London, UK; St George’s University of London, The Vaccine Institute, London, UK; St George’s University of London, Paediatric Infectious Diseases Research Group, London, UK; Oxford Vaccine Group, Department of Paediatrics, University of Oxford, UK; Oxford Vaccine Group, Department of Paediatrics, University of Oxford, UK; St George’s University of London, Paediatric Infectious Diseases Research Group, London, UK; St George’s University of London, Paediatric Infectious Diseases Research Group, London, UK; UnivLyon, Claude Bernard University Lyon I, France; St George’s University of London, The Vaccine Institute, London, UK; St George’s University of London, The Vaccine Institute, London, UK; St George’s University of London, Paediatric Infectious Diseases Research Group, London, UK; St George’s University of London, Paediatric Infectious Diseases Research Group, London, UK; St George’s University of London, The Vaccine Institute, London, UK; St George’s University of London, The Vaccine Institute, London, UK; Imperial College London, Department of Infectious Disease, London, UK; St George’s University of London, Paediatric Infectious Diseases Research Group, London, UK; Makerere University John Hopkins Research Collaboration, Kampala, Uganda; Pathogen Immunology Group, United Kingdom Health Security Agency, Porton Down, UK

**Keywords:** group B *Streptococcus*, neonatal infection, colonization, mucosal immunity, capsular-polysaccharides-specific antibodies, GBS vaccine

## Abstract

Group B *Streptococcus* (GBS) is a leading cause of adverse pregnancy outcomes due to invasive infection. This study investigated longitudinal variation in GBS rectovaginal colonization, serum and vaginal GBS capsular polysaccharide (CPS)-specific antibody levels. Non-pregnant women were recruited in the UK and were sampled every 2 weeks over a 12-week period. GBS isolates were taken from recto-vaginal swabs and serotyped by polymerase chain reaction. Serum and vaginal immunoglobulin G (IgG) and nasal immunoglobulin A (IgA) specific to CPS were measured by Luminex, and total IgG/A by ELISA. Seventy women were enrolled, of median age 26. Out of the 66 participants who completed at least three visits: 14/47 (29.8%) women that were GBS negative at screening became positive in follow-up visits and 16/19 (84.2%) women who were GBS positive at screening became negative. There was 50% probability of becoming negative 36 days after the first positive swab. The rate of detectable GBS carriage fluctuated over time, although serum, vaginal, and nasal CPS-specific antibody levels remained constant. Levels of CPS-specific antibodies were higher in the serum of individuals colonized with GBS than in non-colonized, but similar in the vaginal and nasal mucosa. We found correlations between antibody levels in serum and the vaginal and nasal mucosa. Our study demonstrates the feasibility of elution methods to retrieve vaginal and nasal antibodies, and the optimization of immunoassays to measure GBS-CPS-specific antibodies. The difference between the dynamics of colonization and antibody response is interesting and further investigation is required for vaccine development.

## Teaser

70 non-pregnant women were recruited, from which 21 were GBS positive at baseline. GBS recto-vaginal colonisation and capsule polysaccharides specific antibody levels in serum, and in the vaginal and nasal mucosa were followed for three months. Colonisation and GBS serotypes present fluctuated over time.

## Graphical Abstract

**Graphical Abstract:**
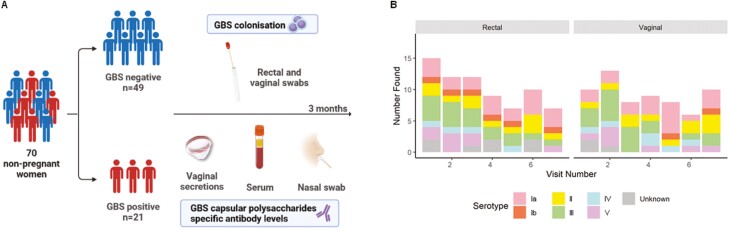

## Introduction

Infection is one of the most important causes of adverse pregnancy outcomes, with group B *Streptococcus* (GBS) a leading cause of invasive neonatal disease, maternal infection, stillbirth, and premature delivery worldwide [1, 2]. The estimated incidence rate of invasive neonatal GBS disease in the UK ranges from 0.94 to 1.28 per 1000 live births [3, 4]. Maternal rectovaginal or urogenital colonization with GBS is a prerequisite for early-onset neonatal disease (within the first 6 days of life), with vertical transmission occurring around the time of birth [5]. Intrapartum antibiotic prophylaxis (IAP) is the only method of disease control currently available [6]. IAP policies are poorly sensitive and specific at identifying neonates with invasive disease, resulting in both missed cases and unnecessary antibiotic administration. For example, a UK study found that 65% of babies who developed a early-onset disease were born to mothers with no sepsis risk factors [3].

Given the shortcomings of IAP strategies, novel methods of GBS control are urgently required. The World Health Organization considers the development of a maternal GBS vaccine a high priority [7, 8], and there are currently several potential candidates [9]. One challenge with establishing vaccine efficacy against invasive neonatal disease as a primary endpoint is the very large number of pregnant women who would need to be recruited to phase 3 studies [10]. Efforts are underway to develop a controlled human infection model (CHIM) for GBS to gain further insight into host–pathogen interactions. A GBS CHIM could provide the opportunity to improve our understanding of immune parameters associated with different clinical outcomes (colonization, disease), and ultimately appropriate serocorrelates of protection. A validated serocorrelate of protection could provide a potential surrogate endpoint for vaccine efficacy studies.

The current study investigated longitudinal variation in GBS rectovaginal colonization, serum and vaginal GBS capsular polysaccharide (CPS)-specific IgG, and CPS-specific IgA response in the nasal mucosa.

## Materials and Methods

### Study design and outcome measures

This was a pilot, prospective observational cohort study conducted at St George’s Hospital in London, UK (trial registry identification NCT04059510). The study was approved by the HRA and HCRW (Health Research Authority and Health and Care Research Wales, IRAS number 261556). The primary outcomes were to determine GBS colonization status of non-pregnant women every two weeks over a 12-week period, and to determine the concentration of CPS-specific antibodies in GBS colonized and non-colonized women: total and CPS-specific IgG in vaginal secretions at baseline and 2-week intervals, CPS-specific IgG in serum at 6-week intervals, and total and CPS-specific IgA in nasal samples at baseline and after 12 weeks. The secondary outcomes were to determine the correlation between GBS antibodies in serum, and in the vaginal and nasal mucosa.

### Recruitment and eligibility

The study was advertised using posters around the hospital and notices on websites. Healthy non-pregnant women aged 18-40 were eligible. Participants had to be willing to use adequate contraception for the duration of the study. Women with any of the following were ineligible: history of latex allergy, current intra-uterine device/intra-uterine system, presence of an untreated genitourinary infection including sexually transmitted infections, diabetes mellitus, genital dermatoses, cervical intraepithelial neoplasia within the past three years, and post-menopausal women. Those found to have a genitourinary infection at screening were eligible for inclusion if the infection was fully treated or appropriately managed prior to commencing the main study.

### Screening

Following written informed consent, volunteers underwent screening by medical staff (nurses, midwives or physicians). The participant’s medical history was discussed (co-morbidities, smoking and alcohol use, and method of contraception). Rectal and low-vaginal Copan Italia SPA swabs (Italy) were taken to determine GBS carriage status. A urine pregnancy test and a genitourinary infection screen were performed for *Chlamydia trachomatis*, *Neisseria gonorrhoeae*, *Trichomonas vaginalis*, Candida species, *Gardnerella vaginalis* (two genital swabs), and a 10-mL venous blood sample was taken for syphilis, human immunodeficiency virus (HIV), and hepatitis B and C. If a participant tested positive for any of these infections, they were referred to a local healthcare provider for treatment if it was deemed necessary.

### Study procedures

Study data were collected and managed using REDCap electronic data capture tools [11, 12].

Any participant meeting the full inclusion criteria following screening was invited to take part in the main study, for twelve weeks with seven visits in total (Supplementary Data). The maximum time between screening and the first visit was 28 days, with each subsequent visit separated by two weeks (minimum 7 days to a maximum of 21 days).

Recto-vaginal swabs for GBS carriage were taken at each visit, and a self-inserted menstrual cup (Softdisc^TM^) to collect vaginal secretions. The vaginal cups remained in position for 1 hour before being removed and frozen. At visits 1, 4 and 7, 10 mL of venous blood was taken by venepuncture, in addition to a urine pregnancy test. At visits 1 and 7, a synthetic absorptive matrix strip (SAM strip, Nasorption^TM^) was placed into participants’ nostrils for 2 minutes to collect nasolacrimal fluid.

### Changes to study procedures because of COVID-19

Social distancing measures were introduced in the UK on 23 March 2020 making in-person study visits impossible. Participants who had completed at least the first visit and who agreed to continue self-sampling at home were provided with the study materials by post. Participants were instructed to triple-pack samples in specimen bags and freeze swabs and vaginal cup immediately following collection in their home freezer. When social distancing measures were lifted, the study team retrieved the specimens with ice packs. Venous blood draws, urine pregnancy tests, and nasolacrimal swabs were unable to be collected.

### Microbiology and serotyping

Rectal and vaginal swabs in Amies gel were delivered to the St George’s Vaccine Institute following collection; during the COVID-19 lockdown they were cut into skim milk, tryptone, glycerol, and glucose (STGG) storage medium by participants and frozen at −20°C at home until collection. Once collected, the swabs were inoculated into Todd-Hewitt Broth with nalidixic acid (15 mg/mL) and colistin (10 mg/mL) (lysine indole motility medium, LIM broth). The LIM was incubated for 6-18 h at 37°C in 5% CO_2_. Ten microliters of this culture were streaked to single colonies on CHROMagar StrepB (CHROMagar, France) and incubated for 18-24 h at 37°C. Positive colonies were selected for subculture on Columbia horse blood agar (Oxoid, England) and incubated for 24-48 h at 37°C in 5% CO_2_. Identity was confirmed using a Matrix Assisted Laser Desorption/Ionization Time-of-Flight Mass Spectrometer (MALDI-TOF MS, MALDI).

Isolates were re-cultured for 18 h in LIM broth, then centrifuged at 5000 × *g* for 10 min. Pellets were resuspended in 200 µL of lysis buffer (1 M Tris–HCl, 0.5 M EDTA, 10% Triton X-100), 40 µL of mutanolysin (3ku), 40 µL of lysozyme (100 mg/mL) and 4 µL of RNase (100 mg/mL) per sample. After incubation at 37°C for 1 h, 20 µL of proteinase K and 200 µL of AL buffer (QIAGEN, Germany) were added to each sample, and incubated 90 min at 56°C.The subsequent steps followed the manufacturer’s instructions, and the last elution step was realized with 100 µL of filtered distilled water. Extracted DNA was quantified using a Qubit fluorometer (Invitrogen, USA).

For serotyping, the extracted DNA was run in a polymerase chain reaction assay (PCR) with 19 primers for GBS CPS genes using the PCR Multiplex Master Mix (Qiagen, Germany). This included a 15-min denaturing step at 95°C, followed by 35 cycles of 95, 54, and 72 for 1, 1, and 2 min, respectively. The amplified product was visualized on a 1.5% agarose gel. 5 µL of each sample was run with a NEB 100bp purple DNA ladder (New England Biolabs, USA), using SYBR Safe (Life Technologies, USA) gel stain, while immersed in tris-acetate EDTA buffer. Serotype standards obtained from Public Health England were used as controls and to assist in identification [13]. Gels were imaged using a Bio-Rad Gel Doc XR+ (Bio-Rad, USA). Positive isolates identified during the screening visit were not serotyped.

### Antibody elution from mucosal samples

Nasal strips were placed into 300 µL of extraction buffer (Dulbecco’s phosphate-buffered saline, 0.25 M NaCl with Calbiochem Protease inhibitor Set I) and left overnight on a shaker at 4°C. Strip were placed in spin columns and centrifuged at 16 000 × *g* for 10 min at 4°C.

Vaginal cups placed in 50 mL tubes were centrifuged at 2500 × *g* for 5 min. Cups were removed and collected secretions weighted. Extraction buffer was added proportionally to the weight of secretion (100 µL for 100 mg) and the mix transferred to small tubes. After centrifugation at 16 000 × *g* for 10 min, supernatant was collected.

### GBS CPS-specific antibody measurement

GBS CPS-specific IgG in serum and vaginal secretions, and CPS-specific IgA in nasal samples, were measured using a multiplex immunoassay. MagPlex microspheres (Luminex Corp) were coated with CPS-poly-l-lysine conjugates for GBS serotypes Ia, Ib, II, III, IV and V (Pfizer). Samples were diluted in assay buffer (0.5% bovine serum albumin on 1× phosphate-buffered saline (PBS), 0.05% Tween-20, 0.02% sodium azide, pH 7.2) to the following dilutions: serum 1:25, 1:250, 1:2500, vaginal secretions 1:5, 1:25, 1:100 and nasal samples 1:10, 1:20, 1:40. Standard curves were made from serially diluted positive serum (supplied by Carol Baker) for IgG, or from pooled positive breastmilk samples for IgA. Breastmilk samples were previously collected from a cohort of Gambian women in 2014 [14], and the study was approved by the joint Gambian Government/Medical Research Council Research Ethics Committee, SCC 1350 V4. Samples and beads were incubated overnight at 4°C. After washing (1× PBS, 0.05% Tween-20), samples were incubated for 1.5 h, with a 1:500 dilution in wash buffer of R-Phycoerythrin-conjugated goat α-human IgG Fcγ-specific (109-115-098, Jackson ImmunoResearch Cambridge UK) or R-Phycoerythrin-conjugated goat α-human α IgA chain Ab (109-115-011 Jackson ImmunoResearch Cambridge UK).

### Total IgG and IgA measurement

Total IgG was measured in vaginal secretions by ELISA. Briefly, plates were coated with Fab-specific anti-human IgG (I5260 Sigma-Aldrich) at 2 µg/mL, then incubated overnight at 4°C. Blocking buffer (1× PBS, 0.5% Tween-20, 1% skimmed milk powder) was added for 3 h. Samples were diluted to 1:40000, 1:80000, 1:160000, 1:320000 in blocking buffer, and run against a standard curve of serially diluted IgG from human serum (I2511 Sigma-Aldrich). Plates were incubated overnight at 4°C, then incubated with 1:100000 anti-human IgG-horseradish peroxidase (A8667 Sigma-Aldrich) for 3 h. 3ʹ,5,5ʹ-Tetramethylbenzidine substrate (TMBW-1000-01, Cambridge bioscience) was added for 5 min, and the reaction stopped with 0.2 M H_2_SO_4_. Optical density was read at 450 nm. Plates were washed between each step with 1× PBS, 0.5% Tween-20.

Total IgA was measured in nasal samples by ELISA, following the protocol of De Silva et al [15].. Samples were diluted 1:40000, 1:80000, 1:160000 and 1:320000 and run against a curve of serially diluted purified human secretory IgA (Bio-Rad PHP133).

### Statistical analysis

No formal sample size calculation was undertaken because this was a pilot study. Mean, median and proportions were used for descriptive analyses. GBS positivity was considered when either the rectal or vaginal swab were positive. Probability of GBS clearance over time was estimated with a Kaplan-Meier analysis.

Antibody concentrations were interpolated using a 5-parameter logistic regression for Luminex assays and a 4-parameter logistic regression for ELISA assays. For serum, CPS-specific IgG concentrations under the limit of quantification (0.1 µg/mL) were assigned a value of 0.05 µg/ml. Vaginal CPS-specific IgG concentrations above the limit of quantification (0.1 µg/ml) were expressed as a proportion of total IgG in ‰, and nasal antibody concentrations as CPS-specific IgA/ total IgA in arbitrary units (AU). For mucosal samples, concentrations falling under the limit of quantification of the Luminex assay were removed to prevent bias during adjustment with the total antibody values.

Serum antibodies results did not follow a normal or log normal distribution therefore non-parametric tests were used: Spearman for correlation, and Mann–Whitney for median comparison between colonized and non-colonized.

Missing data were assumed missing completely at random with respect to the response variables and excluded from the analysis. A 5% significance threshold was set. Analyses were completed using STATA version 17 [16], Bio-Plex Manager Software version 6.2, GraphPad Prism version 9.0.0 [17], and R version 4.1.1 [18].

## Results

### Screening and participant flow

**Figure 1:**
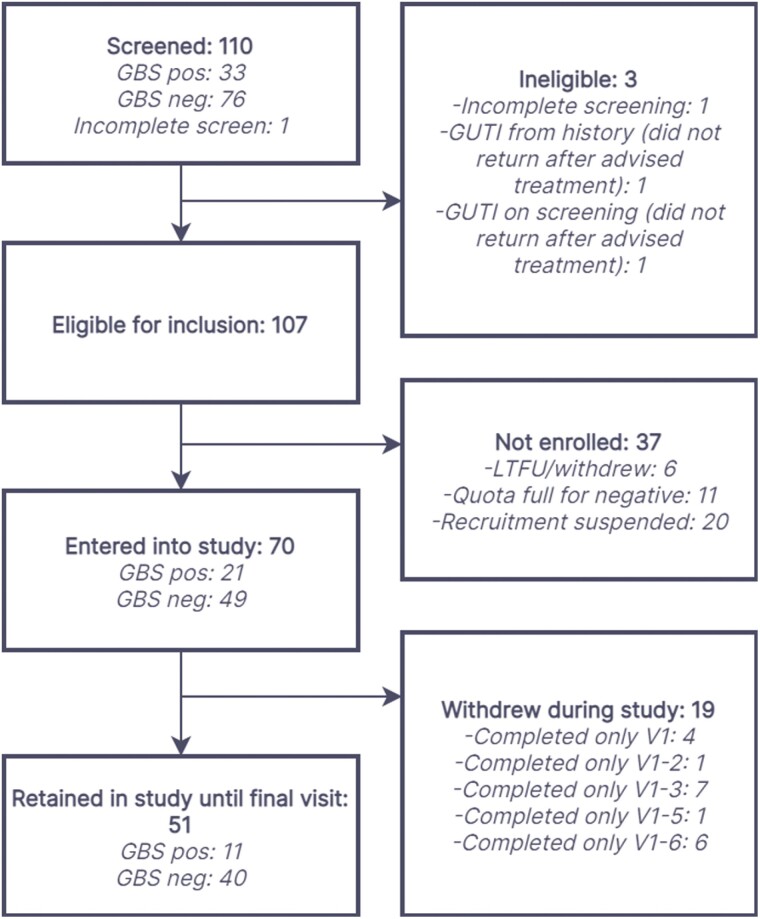
flow of participants through study. LTFU: lost to follow-up; GUTI: genitourinary infection.

Between November 2019 and March 2020, 110 women underwent screening, with 107 eligible for inclusion and 70 entering the main study (Figure 1). Of these 70 participants, there were 51 who remained in the study until the final visit, with 47 completing all 7 visits and 66 completing at least three visits.

### Demographic details

The median age of the 51 women completing the study was 26 years (IQR 24-30), and 11 (21.6%) were GBS positive at baseline (Table 1). The majority of participants were never-smokers (40/51, 78.4% for cigarettes and 49/51, 96.1% for e-cigarettes) and consumed one to five alcoholic beverages per week (38/51, 74.5%). The most common methods of contraception were the combined oral contraceptive pill (18/51, 35.3%) and condoms/diaphragm (17/51, 33.3%). Of women completing the study, 4/51 (7.8%) and 4/51 (7.8%) were positive or had an intermediate bacterial vaginosis result respectively, and 3/51 (5.9%), 3/51 (5.9%), and 1/51 (2.0%) had positive, intermediate, or mild candida screens, respectively. There were no important differences in the baseline demographic details between those who underwent screening and those who completed the study (Table 1).

**Table 1:** baseline demographics of participants

		Participants screened (*n* = 110)n (%)	Participants completing study (*n* = 51)n (%)
**Age [median (IQR)]**		26 (23-29)	26 (24-30)
**GBS**	Positive	33 (30.0)	11 (21.6)
	Negative	76 (69.1)	40 (78.4)
	Missing	1 (0.9)	0 (0.0)
**Cigarette smoking**	Current	12 (10.9)	4 (7.8)
	Ex-smoker	16 (14.6)	6 (11.8)
	Never smoker	80 (72.7)	40 (78.4)
	Missing	2 (1.8)	1 (2.0)
**Cigarettes per day [median (IQR)]**		2 (1-3)	1 (1-3)
**E-cigarette use**	Current	2 (1.8)	1 (2.0)
	Ex-smoker	2 (1.8)	0 (0.0)
	Never smoker	104 (94.6)	49 (96.1)
	Missing	2 (1.8)	1 (2.0)
**Alcohol consumption (drinks/week)**	None	7 (6.4)	3 (5.9)
	1–5	78 (70.9)	38 (74.5)
	6–10	22 (20.0)	9 (17.7)
	11–15	1 (0.9)	1 (2.0)
	≥16	0 (0.0)	0 (0.0)
	Missing	2 (1.8)	0 (0.0)
**Current contraception**	Combined oral contraceptive pill	35 (31.8)	18 (35.3)
	Progesterone only pill	12 (10.9)	2 (3.9)
	Abstinence from sex	17 (15.5)	7 (13.7)
	Condom/diaphragm	30 (27.3)	17 (33.3)
	Sex only with other women	2 (1.8)	0 (0.0)
	Implant	9 (8.2)	4 (7.8)
	Other	4 (3.6)	3 (5.9)
	Missing	1 (0.9)	0 (0.0)
***Chlamydia trachomatis***	Positive	0 (0.0)	0 (0.0)
	Negative	109 (99.1)	51 (100.0)
	Missing	1 (0.9)	0 (0.0)
***Neisseria gonorrhoea***	Positive	1 (0.9)	0 (0.0)
	Negative	108 (98.2)	51 (100.0)
	Missing	1 (0.9)	0 (0.0)
***Trichomonas vaginalis***	Positive	0 (0.0)	0 (0.0)
	Negative	109 (99.1)	51 (100.0)
	Missing	1 (0.9)	0 (0.0)
***Gardnerella vaginalis***	Positive	9 (8.2)	4 (7.8)
	Intermediate	8 (7.3)	4 (7.8)
	Negative	92 (83.6)	43 (84.3)
	Missing	1 (0.9)	0 (0.0)
**Candida species**	Positive	6 (5.5)	3 (5.9)
	Intermediate	5 (4.6)	3 (5.9)
	Mild	10 (9.1)	1 (2.0)
	Negative	88 (80.0)	44 (86.3)
	Missing	1 (0.9)	0 (0.0)
**HIV**	Positive	0 (0.0)	0 (0.0)
	Negative	109 (99.1)	51 (100.0)
	Missing	1 (0.9)	0 (0.0)
**Hepatitis B**	Positive	0 (0.0)	0 (0.0)
	Negative	109 (99.1)	51 (100.0)
	Missing	1 (0.9)	0 (0.0)
**Hepatitis C**	Positive	0 (0.0)	0 (0.0)
	Negative	109 (99.1)	51 (100.0)
	Missing	1 (0.9)	0 (0.0)
***Treponema pallidum***	Positive	0 (0.0)	0 (0.0)
	Negative	109 (99.1)	51 (100.0)
	Missing	1 (0.9)	0 (0.0)

GBS: group B *Streptococcus*; HIV: human immunodeficiency virus.

### Rectovaginal carriage

Women were considered GBS positive when GBS was detected on rectal and/or vaginal swabs. Of the 70 enrolled participants, 35 (50.0%) were colonized at least during one visit including the screening visit, and 26 (37.1%) when excluding the screening visit. The prevalence of rectal and vaginal GBS detection fluctuated between visits (Table 2). The use of rectal and vaginal swabs, compared with vaginal swab alone, increased GBS detection by an additional mean proportion across visits of 58.3%. A minority of women were positive on both rectal and vaginal swabs (mean proportion across each visit of 40.7%). There was no clear difference in the prevalence of women who were detected as being GBS positive at each visit if only rectal or only vaginal swabs were used (Supplementary Data).

**Table 2:** prevalence of GBS colonization during each study visit

Site of colonization	Screening *n* = 70	Visit 1 *n* = 70	Visit 2 *n* = 63	Visit 3 *n* = 63	Visit 4 *n* = 56	Visit 5 *n* = 58	Visit 6 *n* = 57	Visit 7 *n* = 51
	*n* (%)							
Rectal only	8 (11.4)	9 (12.9)	3 (4.8)	7 (11.1)	5 (8.9)	3 (5.2)	6 (10.5)	0 (0.0)
Vaginal only	5 (7.1)	4 (5.7)	4 (6.3)	2 (3.2)	6 (10.7)	4 (6.9)	2 (3.5)	3 (5.9)
Both rectal and vaginal	8 (11.4)	6 (8.6)	10 (15.9)	5 (7.9)	3 (5.4)	4 (6.9)	4 (7.01)	7 (13.7)
Rectal and/or vaginal	21 (30.0)	19 (27.1)	17 (27.0)	14 (22.2)	14 (25.0)	11 (19.0)	12 (21.1)	10 (19.6)

Proportions are of those non-missing per visit. GBS: group B *Streptococcus.*

**Figure 2:**
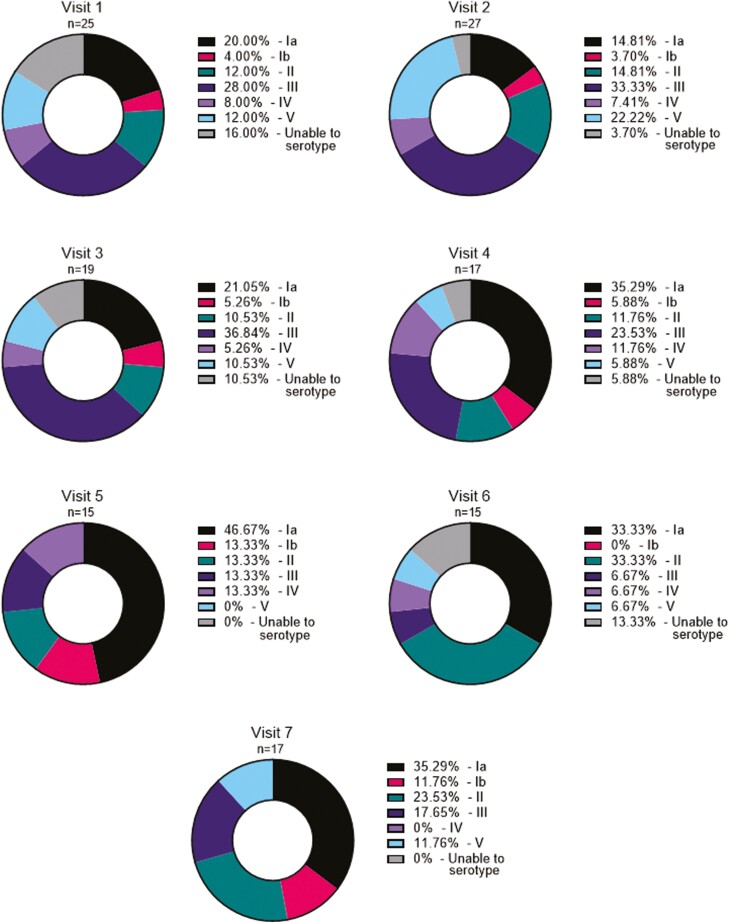
proportion of GBS serotypes, by visit, detected by rectal and vaginal swabs. Percent serotype calculated using the total number of isolates at each visit as the denominator.

There were 136 GBS isolates identified, of which 10 were not able to be serotyped (Supplementary Data). The most common cumulative serotype across the study was serotype Ia (37/136, 27.2%), followed by III (34/136, 25.0%), II (22/136, 16.2%), V (15/136, 11.0%), IV (10/136, 7.4%), and Ib (8/136, 5.9%) (Figure 2). Across visits, eight individuals were colonized with serotype Ia, eight with III, six with II, three with IV, three with V, and one with Ib (Supplementary Data). Four individuals carried more than one serotype overtime, and three individuals were colonized vaginally and rectally with different serotypes at the same visit.

Dynamics in colonization are detailed in Supplementary Data. From 66 participants (94.3%) who completed three or more study visits: 33 (50.0%) were persistently negative throughout the study, and three (4.5%) were persistently positive. Four (6.1%) were positive at one visit only, with the remaining visits GBS negative, and a further four were negative only once during the study with the remaining visits GBS positive. 11 participants (16.7%) moved between positivity and negativity. Nine participants (13.6%) who were initially positive became and remained negative. Two participants (3.0%) were initially negative, then became and remained positive.

**Figure 3:**
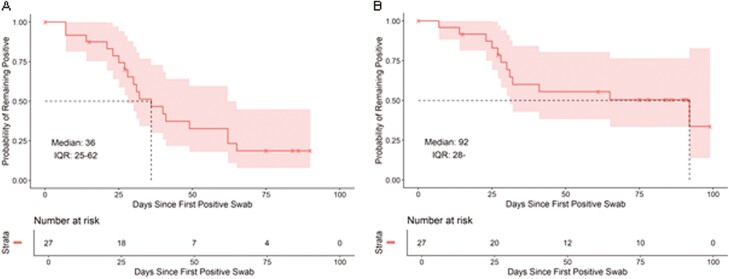
estimated time before rectovaginal GBS clearance. Estimated with the Kaplan-Meier method, including 95% confidence bands. Time from the first positive to the first negative swab (A), and time from the first positive swab to clearance until the end of the study (B). The upper bound of IQR in the right-hand panel is out of data range and not computable.

When considering the time between the first positive and first negative swab, the probability of remaining positive dropped to 50% after 36 days (Figure 3A). However, when considering participants that cleared GBS by the end of the study, there was still a 50% chance of being colonized after 92 days (Figure 3B).

Of the 22 individuals taking the combined oral contraceptive pill and completing the study, nine (40.9%) were colonized with GBS at one or multiple time points, and 13 (59.1%) remained non-colonized throughout. Of the five individuals taking progesterone only pill and completing the study, two (40%) were colonized with GBS at multiple time points, three (60%) remained non-colonized.

### Antibody evolution over time

The total number of specimens collected per site is displayed in Supplementary Data and the distribution of measured antibodies in Supplementary Data.

**Figure 4:**
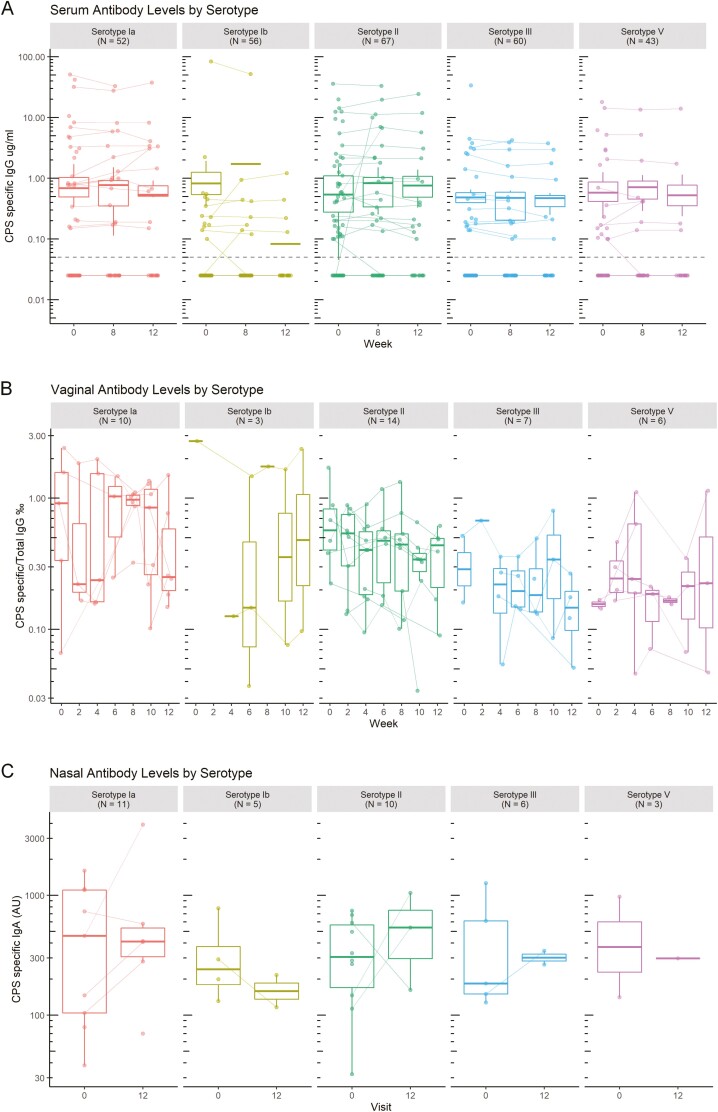
timecourse of CPS-specific antibody levels. CPS-specific IgG levels were measured in serum (A) and vaginal secretions (B), and IgA in nasal samples (C) over the 3 months of the study. Serum was collected on weeks 0, 6, and 12. Vaginal secretions were collected every 2 weeks. Nasal samples were collected on weeks 0 and 12. The concentration of GBS CPS-specific antibodies were measured at each time point. Concentrations on log scale. Each line represents an individual. Medians and quartiles are plotted.

GBS CPS-specific antibody levels were investigated over the 3 months of the study. Out of the 70 participants, serum CPS-specific IgG were over 0.1 µg/mL in 23 (32.9%) individuals for serotype Ia, 14 (20%) for Ib, 44 for II (62.9%), 20 (28.6%) for III and 19 (27.1%) for V. Five individuals did not have quantifiable CPS-specific serum antibodies. For seropositive individuals, levels were largely constant over time for all serotypes (Figure 4A) and there was no change in titre in individuals colonized with the matching strain.

CPS-specific IgG were measurable in vaginal secretions from 27/70 women (Figure 4B). In the small numbers of vaginal samples with CPS-specific IgG, the levels reflected the serum data, with a relatively constant level of response throughout the study. This was no different in the individuals colonized compared to those who were not colonized.

CPS-specific IgA in nasal samples were measured in 23/70 individuals, however only nine completed the nasal swabbing at the first and last visits. Nasal CPS-specific IgA levels (Figure 4C) were constant over the 3 months of the study.

There was no visible change of antibody levels over the course of the study in serum, or in the vaginal and nasal mucosa. Antibody levels were not related to gain or loss of GBS colonization. Moreover, after GBS clearance, CPS-specific antibody levels remained constant for at least 3 months.

### The impact of GBS colonization on antibody levels

**Figure 5:**
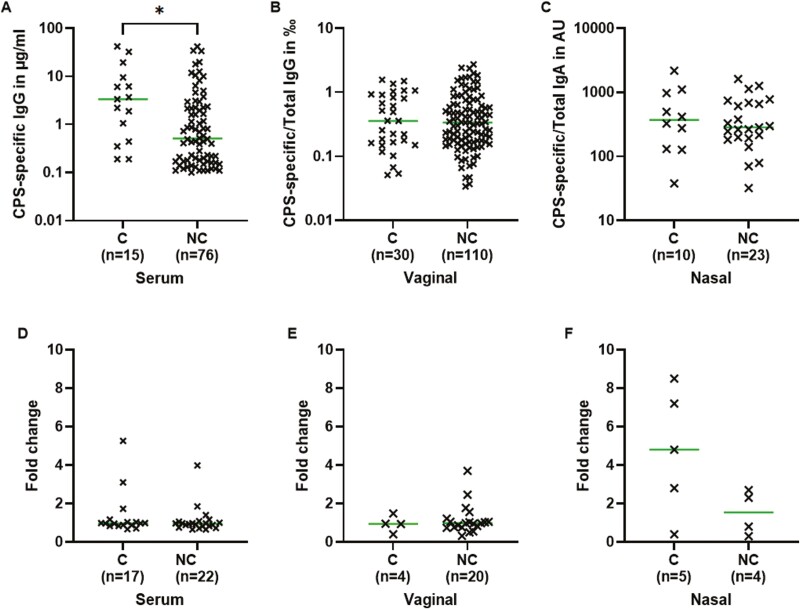
CPS-specific antibodies between colonized (C) and non-colonized (NC) individuals in serum and nasal and vaginal mucosa. An individual was defined as colonized with a serotype if they carried this serotype at one or more visits. Concentrations plotted are, for each individual, the average concentrations between all visits. Medians compared with a Mann-Whitney test in serum (A), vaginal (B), and nasal samples (C), concentration on log scale. Fold change in serum (D), vaginal (E), and nasal samples (F) was calculated between levels measured in the last collected sample over the first collected sample for each individual. Concentrations of serum CPS-specific IgG in µg/ml, of vaginal CPS-specific IgG/total IgG in ‰ and of nasal CPS-specific IgA/total IgA in AU.

Participants were split into two groups: women with CPS-specific antibodies that were colonized with the same serotype at any time in the study (C) and women either non-colonized by GBS or colonized with another serotype (NC). As antibody levels were constant over time, the average concentration between visits was used for this analysis. The median for CPS-specific IgG was 6.8 times higher in the serum of colonized individuals than in non-colonized individuals (Figure 5A, Mann–Whitney, *P*-value = 0.01). There was no difference in the levels of CPS-specific IgG in vaginal samples (Figure 5B, Mann–Whitney, *P*-value = 0.89) or in the levels of CPS-specific IgA in the nasal mucosa (Figure 5C, Mann–Whitney, *P*-value = 0.74). There was no difference in fold change in serum, vaginal, and nasal antibody levels between colonized and non-colonized women (Figure 5D-F).

**Figure 6:**
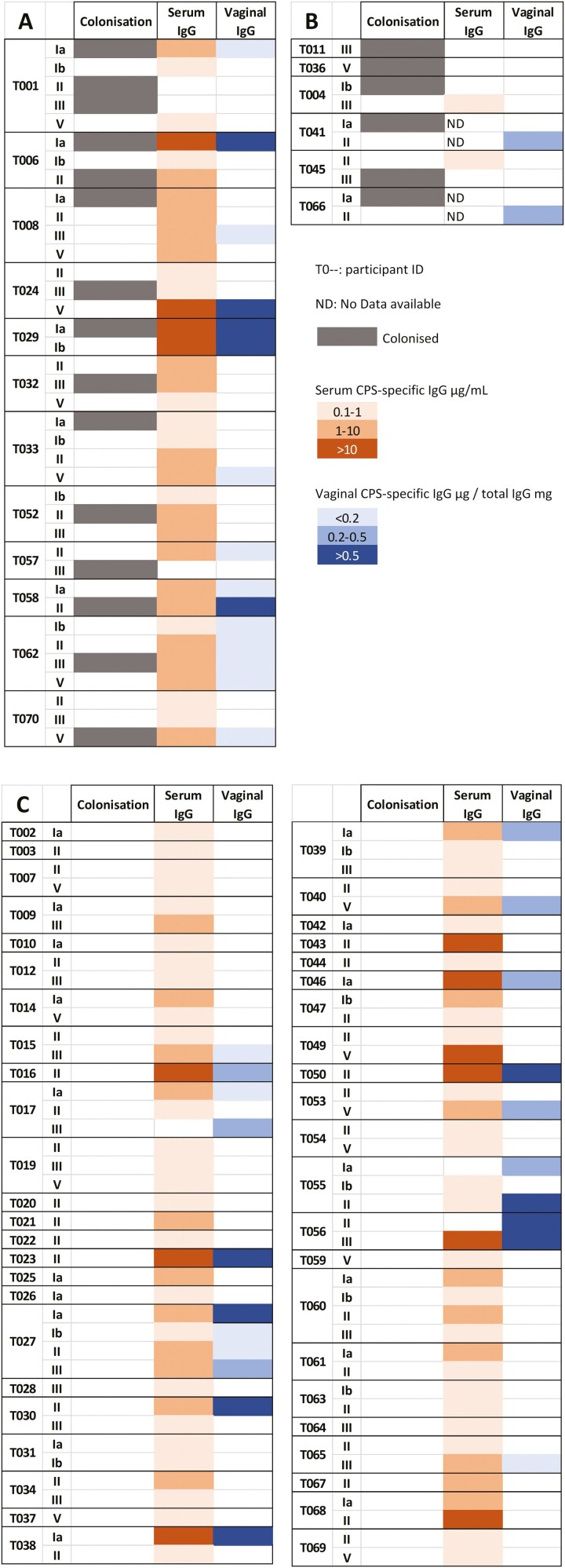
colonization status and CPS-specific antibody levels. Average concentrations between all visits, only concentrations above the assay limit of quantification of 0.1 µg/ml are displayed. A: Colonized individuals with homologous antibodies. B: Colonized individuals with GBS CPS-specific antibodies for heterologous serotypes. C: Non-colonized individuals with GBS CPS-specific antibodies.

Seventeen percentage of the participants were colonized with GBS and exhibited homologous antibodies to the serotype of colonization (Figure 6A). Some individuals (9%) were colonized with GBS but had no detectable antibody for the colonizing serotype (Figure 6B). The majority of individuals (62.9%) were not colonized by GBS during the study visits; however, they exhibited detectable antibody levels in both the serum and vagina, for various GBS serotypes (Figure 6C).

### Antibody responses correlate in serum and in the vaginal and nasal mucosa

**Figure 7:**
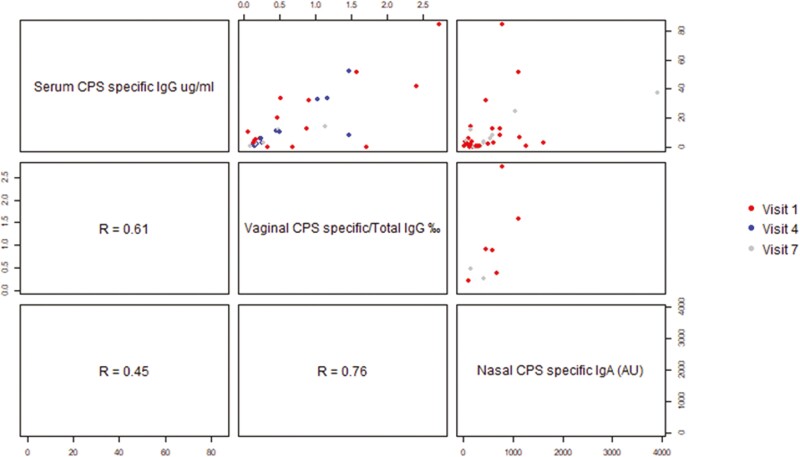
correlation between CPS-specific antibody concentrations in serum, vaginal secretions, and nasal samples. Spearman correlation coefficient are respectively, between serum and vaginal secretions: *r* = 0.61 (*n* = 32), between vaginal secretions and nasal samples: *r* = 0.76 (*n* = 8), and between serum and nasal samples: *r* = 0.45 (*n* = 37).

Out of 27 individuals with measurable vaginal CPS-specific IgG, a significant correlation was found between the level of vaginal and serum CPS-specific IgG for 19 individuals (Figure 7, *r* = 0.61), for a total of 32 visits. However, for some individuals, CPS-specific IgG was measurable in vaginal secretions but not in serum (Figure 6).

Five individuals had matching nasal CPS-specific IgA and vaginal IgG that were significantly correlated (Figure 7, *r* = 0.76), for a total of eight visits.

### Colonizing serotype distribution and frequency of CPS-specific antibodies

**Figure 8:**
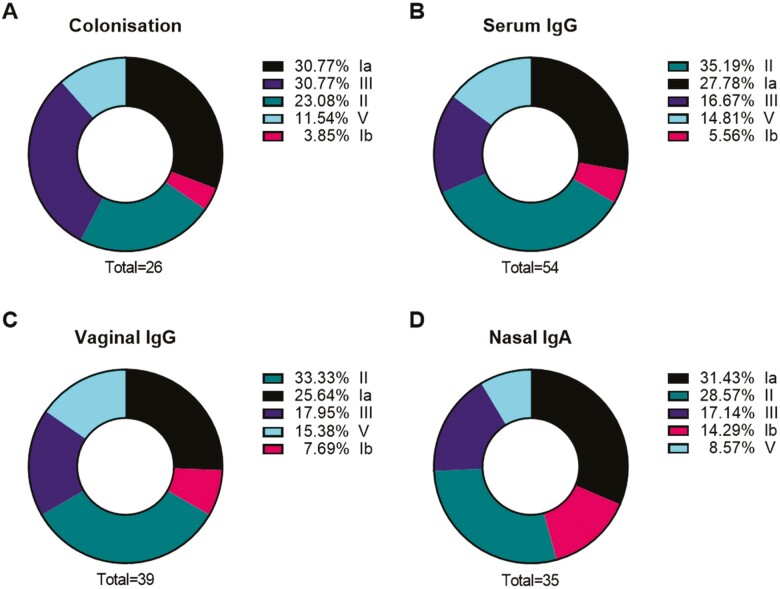
proportion of colonizing serotypes (A), and proportions of CPS-specific antibodies in serum (B) and vaginal (C) and nasal mucosa (D). An individual was defined as colonized or as having serotype-specific antibody if detection occurred at least once during study visits. Some individuals carried multiple serotypes and had multiple different CPS-specific antibodies detected. The denominator for proportions is the total number of women who were colonized (A) or with detected antibody by site (B, C, D).

The prevalence of serotypes of colonization (Figure 8A) was similar to serotype-specific antibody distribution in serum and in the vaginal and nasal mucosa (Figure 8B, C, D), with the three most common serotypes being Ia, II and III, and the least common Ib and V.

## Discussion

We demonstrated that we are able to elute and measure GBS CPS-specific antibody in different bodily fluids: serum, vaginal, and nasal samples for the first time, and to explore the dynamic interplay between those antibodies and colonization of the rectovaginal tract, with a view to developing a mucosal vaccine.

GBS colonization was variable across the participants. Whilst 62.9% of participants remained negative throughout the study, others were more variable with some evidence of either strain replacement or co-colonization. There was no clear boosting effect of colonization on antibodies in serum and in the vaginal and nasal mucosa, with GBS CPS-specific antibodies remaining relatively stable over the 3 months of the study. Changes in colonization over a short-time period may cause positive cases to be missed while screening for GBS in pregnancy. The relative stability of the antibody response could imply that vaccination in the last 3 months of gestation would give a persistent protective antibody level to lower the risk of early-onset disease [19–21].

We found that levels of CPS-specific IgG were 6.8 times higher in the serum of colonized individuals than non-colonized individuals, but similar in the vagina. Of note, a study showed that higher serum antibody levels were associated with a lower risk of GBS acquisition during pregnancy [22]. Conversely, another study of pregnant women rectally or vaginally colonized with GBS showed higher serotype-specific IgG and IgA levels in cervical secretions than in non-colonized women, but similar levels in serum [23].

A correlation between vaginal and serum CPS-IgG was found regardless of colonization status, supporting the hypothesis that vaginal IgG are a transudate from serum [24–26] and could be induced by parenteral vaccination [27]. However, the presence of quantifiable CPS-specific IgG in the vagina but not in the serum of three individuals suggests that there is also local mucosal IgG production, as it has been suggested in the cervix for GBS-specific antibodies [28], and HIV-specific antibodies [29]. This indicates that both an intramuscular vaccine or a mucosal vaccine could allow an increase in vaginal IgG and protection against GBS colonization and invasive disease. Enhancing vaginal antibody responses may help protect the female genital tract, thus diminishing the risk of ascending GBS colonization in the cervix and vertical transmission to the infant.

We have shown that nasal and vaginal antibody responses were correlated, suggesting that intranasal delivery of a vaccine may enhance vaginal antibody levels. It has been observed that GBS intranasal immunization of mice triggered specific IgG and IgA responses in the vagina [30–33], and protected pups against a GBS peritoneal challenge [31]. The first human studies investigating the intranasal route of immunizations were realized with the cholera toxin B (CTB) and successfully elicited distant-specific antibody responses in the vaginal mucosa [34–36]. Currently, a vaccine in clinical trial is looking at the association of intramuscular and intranasal administration to protect against *Chlamydia trachomatis*, another vaginal pathogen [37], and there is a further clinical trial with an intranasal vaccine against the intestinal pathogen *Shigella flexneri* [38]. Our data indicate that a similar mode of administration might be useful for GBS.

### Limitations

Our study has several limitations. The COVID pandemic impacted recruitment of participants with only 70 women recruited, and collection of samples with notable loss of follow-up, missing visits, and potentially variable home storage conditions. Therefore, the number of collected mucosal samples and the levels of antibody detected were relatively limited, which may impact the power of the analysis.

The laboratory capacity only enabled us to serotype one bacterial colony per swab; however, we believe that rectovaginal co-colonization by multiple serotypes is not excluded, as multiple carriage was demonstrated in 6.6% of pregnant women carrying GBS in England [39] and 6.6% of non-pregnant women carrying GBS in the USA [40].

Due to the lack of standardized reagents with known concentrations of IgG or IgA directed against GBS, concentrations for CPS-specific antibodies were interpolated from serially diluted sera (Carol Baker) and breastmilk samples from GBS-positive individuals. However, work is ongoing to develop standardized assays to assess antibody responses to GBS and serocorrelates of protection [41].

## Conclusion

Our study demonstrated that we are able to successfully elute vaginal and nasal antibodies, to measure GBS capsular polysaccharides-specific IgG and IgA with a multiplex immunoassay. These methods may be useful for the development of a mucosal vaccine against GBS, and human challenge models.

## Supplementary Material

uxac066_suppl_Supplementary_Material

## Data Availability

Data are available in the St George’s Research Data Repository (https://sgul.figshare.com).

## Abbreviations

CHIM: controlled human infection model
CPS: capsular polysaccharides
EDTA: ethylenediaminetetraacetic acid
ELISA: enzyme-linked immunosorbent assay
GUTI: genitourinary infection
HRA and HCRW: Health Research Authority and Health and Care Research Wales
HIV: human Immunodeficiency virus
IAP: intrapartum antibiotic prophylaxis
IgG/A: immunoglobulin G or A
IQR: interquartile range
GBS: group B *Streptococcus*
GUTI: genitourinary tract infection
LFTU: lost to follow-up
LIM: lysine indole motility medium
MALDI-TOF MS: matrix-assisted laser desorption/ionisation time-of-flight mass spectrometer
PBS: phosphate-buffered saline
PCR: polymerase chain reaction
STGG: skim milk: tryptone: glucose: and glycerine medium

## Clinical trial registration

Trial registry identification NCT04059510 (www.clinicaltrials.gov).
