# Supplementary material for: Group B Streptococcus (GBS) colonization is dynamic over time, whilst GBS capsular polysaccharides-specific antibody remains stable
Source: Clin Exp Immunol. 2022 Jul 8;209(2):188–200. doi: 10.1093/cei/uxac066 (PMC9390841; doi:10.1093/cei/uxac066)
Supplement: uxac066_suppl_Supplementary_Material [file uxac066_suppl_supplementary_material.docx]

| **Supplementary information 1:** Study procedures at each visit. | | | | | | | | |
| --- | --- | --- | --- | --- | --- | --- | --- | --- |
|  | **Screening** (day -28) | **Visit 1**  (day 0) | **Visit 2**  (day 14±7) | **Visit 3**  (day 28±7) | **Visit 4**  (day 42±7) | **Visit 5**  (day 56±7) | **Visit 6**  (day 70±7) | **Visit 7**  (day 84±7) |
| Participant medical history | x |  |  |  |  |  |  |  |
| STI screening (swabs, blood) | x |  |  |  |  |  |  |  |
| Urine pregnancy testing | x | x |  |  | x |  |  | x |
| Vaginal and rectal swabs for GBS | x | x | x | x | x | x | x | x |
| Menstrual cup for mucosal antibody |  | x | x | x | x | x | x | x |
| Blood draw for serology |  | x |  |  | x |  |  | x |
| Nasolacrimal swab |  | x |  |  |  |  |  | x |
| STI: sexually transmitted infection; GBS: Group B Streptococcus. | | | | | | | | |

| **Supplementary information 2**: Difference in prevalence of GBS positive women, by visit, detected from vaginal only or rectal only swabs. | | | | | | | | | |
| --- | --- | --- | --- | --- | --- | --- | --- | --- | --- |
| **Swab site** | **Screening**  n=70 | **Visit 1**  n=70 | **Visit 2**  n=63 | **Visit 3**  n=63 | **Visit 4**  n=56 | **Visit 5**  n=58 | **Visit 6** n=57 | **Visit 7**  n=51 |
| Number of women with GBS on rectal swab only | 16 | 15 | 13 | 12 | 8 | 7 | 10 | 7 |
| Number of women with GBS on vaginal swab only | 13 | 10 | 14 | 7 | 9 | 8 | 6 | 10 |
| Proportion GBS positive on rectal only swab (%) | 22.9 | 21.4 | 20.6 | 19.0 | 14.3 | 12.1 | 17.5 | 13.7 |
| Proportion GBS positive on vaginal only swab (%) | 18.6 | 14.3 | 22.2 | 11.1 | 16.1 | 13.8 | 10.5 | 19.6 |
| Difference in proportions (%) | 4.3 | 7.1 | -1.6 | 7.9 | -1.8 | -1.7 | 7.0 | -5.9 |
| GBS: Group B *Streptococcus.* | | | | | | | | | |

| **Supplementary information 3:** Number and proportions of colonising GBS serotypes by swab site and visit. | | | | | | | | |
| --- | --- | --- | --- | --- | --- | --- | --- | --- |
| **Serotype** | **Site** | **Number of isolates per visit** | | | | | | |
|  |  | **Visit 1**  n=25 | **Visit 2**  n=27 | **Visit 3**  n=19 | **Visit 4**  n=17 | **Visit 5**  n=15 | **Visit 6**  n=16 | **Visit 7**  n=17 |
|  |  | **n (%)^** | | | | | | |
| Ia | Rectal only | 2 (8.0) | 0 (0.0) | 0 (0.0) | 2 (11.8) | 0 (0.0) | 2 (12.5) | 0 (0.0) |
|  | Vaginal only | 1 (4.0) | 0 (0.0) | 0 (0.0) | 2 (11.8) | 3 (20) | 0 (0.0) | 1 (5.9) |
|  | Rectal and vaginal | 2 (8.0) | 4 (14.8) | 4 (21.1) | 2 (11.8) | 4 (26.7) | 3 (18.8) | 5 (29.4) |
|  | Total (%) | 5 (20.0) | 4 (14.8) | 4 (21.1) | 6 (35.3) | 7 (46.7) | 5 (31.3) | 6 (35.3) |
| Ib | Rectal only | 1 (4.0) | 1 (3.7) | 1 (5.3) | 1 (5.9) | 0 (0.0) | 0 (0.0) | 0 (0.0) |
|  | Vaginal only | 0 (0.0) | 0 (0.0) | 0 (0.0) | 0 (0.0) | 0 (0.0) | 0 (0.0) | 0 (0.0) |
|  | Rectal and vaginal | 0 (0.0) | 0 (0.0) | 0 (0.0) | 0 (0.0) | 2 (13.3) | 0 (0.0) | 2 (11.8) |
|  | Total (%) | 1 (4.0) | 1 (3.7) | 1 (5.3) | 1 (5.9) | 2 (13.3) | 0 (0.0) | 2 (11.8) |
| II | Rectal only | 1 (4.0) | 1 (3.7) | 0 (0.0) | 1 (5.9) | 1 (6.7) | 1 (6.3) | 0 (0.0) |
|  | Vaginal only | 0 (0.0) | 1 (3.7) | 0 (0.0) | 1 (5.9) | 1 (6.7) | 0 (0.0) | 2 (11.8) |
|  | Rectal and vaginal | 2 (8.0) | 2 (7.4) | 2 (10.5) | 0 (0.0) | 0 (0.0) | 4 (25) | 2 (11.8) |
|  | Total (%) | 3 (12.0) | 4 (14.8) | 2 (10.5) | 2 (11.8) | 2 (13.3) | 5 (31.3) | 4 (23.5) |
| III | Rectal only | 2 (8.0) | 1 (3.7) | 1 (5.3) | 0 (0.0) | 2 (13.3) | 1 (6.3) | 0 (0.0) |
|  | Vaginal only | 1 (4.0) | 2 (7.4) | 2 (10.5) | 0 (0.0) | 0 (0.0) | 1 (6.3) | 0 (0.0) |
|  | Rectal and vaginal | 4 (16.0) | 6 (22.2) | 4 (21.1) | 4 (23.5) | 0 (0.0) | 0 (0.0) | 3 (17.6) |
|  | Total (%) | 7 (28.0) | 9 (33.3) | 7 (36.8) | 4 (23.5) | 2 (13.3) | 2 (12.5) | 3 (17.6) |
| IV | Rectal only | 0 (0.0) | 0 (0.0) | 1 (5.3) | 0 (0.0) | 0 (0.0) | 0 (0.0) | 0 (0.0) |
|  | Vaginal only | 0 (0.0) | 0 (0.0) | 0 (0.0) | 2 (11.8) | 0 (0.0) | 1 (6.3) | 0 (0.0) |
|  | Rectal and vaginal | 2 (8.0) | 2 (7.4) | 0 (0.0) | 0 (0.0) | 2 (13.3) | 0 (0.0) | 0 (0.0) |
|  | Total (%) | 2 (8.0) | 2 (7.4) | 1 (5.3) | 2 (11.8) | 2 (13.3) | 1 (6.3) | 0 (0.0) |
| V | Rectal only | 1 (4.0) | 0 (0.0) | 2 (10.5) | 0 (0.0) | 0 (0.0) | 0 (0.0) | 0 (0.0) |
|  | Vaginal only | 0 (0.0) | 0 (0.0) | 0 (0.0) | 1 (5.9) | 0 (0.0) | 1 (6.3) | 0 (0.0) |
|  | Rectal and vaginal | 2 (8) | 6 (22.2) | 0 (0.0) | 0 (0.0) | 0 (0.0) | 0 (0.0) | 2 (11.8) |
|  | Total (%) | 3 (12.0) | 6 (22.2) | 2 (10.5) | 1 (5.9) | 0 (0.0) | 1 (6.3) | 2 (11.8) |
| Unable to serotype | Rectal only | 2 (8.0) | 0 (0.0) | 2 (10.5) | 1 (5.9) | 0 (0.0) | 2 (12.5) | 0 (0.0) |
|  | Vaginal only | 2 (8.0) | 1 (3.7) | 0 (0.0) | 0 (0.0) | 0 (0.0) | 0 (0.0) | 0 (0.0) |
|  | Rectal and vaginal | 0 (0.0) | 0 (0.0) | 0 (0.0) | 0 (0.0) | 0 (0.0) | 0 (0.0) | 0 (0.0) |
|  | Total (%) | 4 (16.0) | 1 (3.7) | 2 (10.5) | 1 (5.9) | 0 (0.0) | 2 (12.5) | 0 (0.0) |
| ^Percent serotype calculated using the total number of isolates at each visit (n) as the denominator. | | | | | | | | |
|  |  |  |  |  |  |  |  |  |
|  |  |  |  |  |  |  |  |  |

Grey box: no sample available .

**Supplementary information 4:** GBS serotypes identified, per individual and by visit.

| **Supplementary information 5:** Number of samples collected. | | | | | | | |
| --- | --- | --- | --- | --- | --- | --- | --- |
| **Specimen** | **Screening**  n=70 | **Visit 1**  n=70 | **Visit 2**  n=63 | **Visit 3**  n=63 | **Visit 4**  n=56 | **Visit 5**  n=58 | **Visit 6** n=57 |
| Rectovaginal swab | 70 | 62 | 64 | 56 | 58 | 57 | 51 |
| Serum | 68 | - | - | 37 | - | - | 23 |
| Vaginal cup | 62 | 62 | 62 | 51 | 54 | 54 | 47 |
| Nasal swab | 70 | - | - | - | - | - | 23 |

‘-‘: not applicable.

**Supplementary information 6:** Distribution of measured antibodies in serum, vaginal and nasal secretions.

GBS capsular polysaccharides (CPS) specific antibodies measured by Luminex, total IgG and IgA measured by ELISA. Serum CPS-specific IgG concentration in ug/ml, vaginal CPS-specific IgG is expressed permille of total IgG (‰) and nasal CPS-specific IgA is expressed in arbitrary units (AU). Measurements for all individuals at all time points are displayed.
